# JAK-STAT Signalling Pathway in Cancer

**DOI:** 10.3390/cancers12071971

**Published:** 2020-07-20

**Authors:** Andrew J. Brooks, Tracy Putoczki

**Affiliations:** 1 The University of Queensland Diamantina Institute, The University of Queensland, Translational Research Institute, Qld 4072, Australia; 2 The Walter and Eliza Hall Institute of Medical Research, Melbourne, VIC 3052, Australia; putoczki.t@wehi.edu.au

JAK-STAT signalling is a cornerstone to cancer progression, either as a tumour intrinsic driver of cancer growth/metastasis, or as a modulator of immune surveillance. This Special Issue highlights new research findings underlying the contribution of JAK-STAT signalling to multiple cancer types, and provides up to date reviews in this critical area from leaders in this field. 

Constitutive activation of JAK-STAT signalling can arise through a number of different mechanisms, including the elevated expression of cytokines. The hallmark example is upregulated interleukin (IL)-6, which signals through either a ‘classic’ mechanism that is restricted by cell-type specific expression of IL-6 receptor α (IL-6Rα), which engages with the ubiquitously expressed β-subunit receptor, GP130. The result is increased Janus kinase (JAK) mediated activation of an oncogenic transcription factor, signal transducer and activator of transcription (STAT)3 [1]. Alternatively, IL-6 ‘trans-signalling’, mediated by IL-6 engagement with a soluble IL-6Rα, can lead to activation of JAK-STAT signalling in any cell [2]. It is now understood that a disintegrin and metalloproteinase 17 (ADAM17) is responsible for the protease-driven shedding of IL-6R [2], revealing a previously unappreciated opportunity to therapeutically modulate IL-6 mediated signalling, with extensive reviews focused on the relevance of IL-6/IL-6R/GP130 to hepatocellular carcinoma and lung cancer provided in this issue [1,2].

Activating mutations in GP130 have been reported that result in ligand independent activation of STAT3 in liver cancers [1]. Activating mutations have also been reported in other cytokine receptors upstream of JAK-STAT, including IL7RA, with a review in this issue providing a new framework for classification of these mutations, based on their structural consequences and biological activity, with oncogenic mutations in acute lymphoblastic leukaemia (ALL) exclusively found in the extracellular and transmembrane regions [3]. While activating mutations in cytokine receptors are not common, or at least not heavily investigated, activating mutations are commonly found in JAK1 and JAK2, which is best highlighted by the literature in myeloproliferative neoplasms and leukaemia. In this issue, a novel JAK1 Y1034C somatic activating mutation was identified from whole genome sequencing of spontaneously occurring rat natural killer large granular lymphocyte leukaemia [4]. Many oncogenic mutations are located in the JAK pseudokinase domain, which is thought to play an essential role in keeping the kinase inactive in absence of ligand binding to a cytokine receptor. This issue includes an enhanced mechanistic understanding of the JAK1 pseudokinase domain in cytokine signalling [5]. Surprisingly, characterisation of the enzyme activity of purified recombinant JAK1 proteins demonstrated that the oncogenic V658F mutation, in the pseudokinase domain, showed no enhancement of intrinsic kinase activity compared to wild-type JAK1 [6]. This study suggests that the structure and interactions of JAK1 in the cellular context are critical for preventing JAK from being activated in the absence of cytokine. 

STAT3 is constitutively activated in numerous cancers. This issue includes multiple new studies investigating the role of STAT3 signalling in several solid malignancies (bladder, breast, medulloblastoma, colon, and cervical cancers), as well as blood cancers (including leukaemia). Activated STAT3 was shown to play a key role in cell viability and invasion of urothelial bladder cancer [7]. In breast cancer stem cells, a novel role of (p21-activated kinase) PAK1 in interaction with JAK2 and STAT3, and in regulating STAT3 signalling was found [8]. Paradoxically, one study revealed low STAT3 expression correlates with improved overall survival in male medulloblastoma patients and identified important sex specific differences in IL-10 and IL-6 expression. In addition, they found that IL-6 enhances SHH-mediated gene regulation in a STAT3-dependent manner [9]. Activated STAT3 was also identified as a potential biomarker for colorectal cancer patients, as peripheral blood T cells from these patients were found to show hyperactivation of STAT3 in response to IL-10 [10]. Moreover, activated STAT3 was reported in the majority of a chronic lymphocytic leukaemia patient cohort, and this level of activation increased with IL-6 or bone marrow mesenchymal stromal cells [11]. A study in cervical cancer cells showed that inhibition of JAK2 reduced STAT3 activation, which correlated with reduced cell proliferation and increased sensitivity to cisplatin [12], highlighting the therapeutic potential of inhibition of this signalling cascade. 

Beyond STAT3, STAT5 is also implicated in cancer progression. The activation of STAT5 can be mediated by growth hormone (GH), which has been shown to upregulate melanocyte-inducing transcription factor (MITF) expression and activity via JAK2-STAT5 in melanoma [13]. STAT5 is also implicated in malignant haematopoiesis, with a detailed review of the contributions of STAT5A and B in this process in this issue [14]. A new genetically engineered mouse model that allows tissue specific combined deletion of STAT3 and STAT5A/B, is also described, and will be invaluable in the study of the role of these signalling proteins in cancer and other diseases [15], since, among other roles, both STAT3 and STAT5 regulate metabolism-related genes [16].

Numerous therapeutics have been, and continue to be, developed to modulate this signalling pathway, with varying degrees of success and failures [17]. Differences in drug targets and efficacy aside, these observations are not entirely surprising, given our emerging understanding of the importance of JAK-STAT signalling in both immune regulation and cancer progression, reviewed within this issue [17]. This issue also includes timely reviews on TYK2 in tumour immunosurveillance [18], the on the role of STAT3 in T and NK cell lymphomas [19], and its involvement in tumour evasion through elevated expression of PD-L1 [20]. STAT3 signalling in CD103^+^ conventional dendritic cells also provides an important immunosuppressive function via IL-10 activation. This study provided further potential for targeting STAT3 for inhibition for improving efficacy of tumour vaccines [21]. 

Despite identification of the JAK-STAT pathway many decades ago, we are only beginning to scratch the surface of our understanding of the intricate details of this signalling cascade in cancer. This special issue provides an overview of our current knowledge in specific cancers and highlights many of the emerging research concepts in the field.
